# In Situ Fabrication of Activated Carbon from a Bio-Waste *Desmostachya bipinnata* for the Improved Supercapacitor Performance

**DOI:** 10.1186/s11671-021-03545-8

**Published:** 2021-05-13

**Authors:** Gopal Krishna Gupta, Pinky Sagar, Sumit Kumar Pandey, Monika Srivastava, A. K. Singh, Jai Singh, Anchal Srivastava, S. K. Srivastava, Amit Srivastava

**Affiliations:** 1 Department of Physics, TDPG College, VBS Purvanchal University, Jaunpur, 222001 India; 2 Department of Physics, Institute of Science, Banaras Hindu University, Varanasi, 221005 India; 3 School of Materials Science and Technology, Indian Institute of Technology (BHU), Varanasi, 221005 India; 4 School of Physical Sciences, Jawaharlal Nehru University, New Delhi, 110067 India; 5 Department of Pure and Applied Physics, Guru Ghasidas Vishwavidyalaya, Bilaspur, 495009 India

**Keywords:** Bio-waste material, Supercapacitor, Electrochemical double-layer capacitance, Kusha grass, Activated carbon, Porosity

## Abstract

Herein, we demonstrate the fabrication of highly capacitive activated carbon (AC) using a bio-waste Kusha grass (*Desmostachya bipinnata*), by employing a chemical process followed by activation through KOH. The as-synthesized few-layered activated carbon has been confirmed through X-ray powder diffraction, transmission electron microscopy, and Raman spectroscopy techniques. The chemical environment of the as-prepared sample has been accessed through FTIR and UV–visible spectroscopy. The surface area and porosity of the as-synthesized material have been accessed through the Brunauer–Emmett–Teller method. All the electrochemical measurements have been performed through cyclic voltammetry and galvanometric charging/discharging (GCD) method, but primarily, we focus on GCD due to the accuracy of the technique. Moreover, the as-synthesized AC material shows a maximum specific capacitance as 218 F g^−1^ in the potential window ranging from − 0.35 to + 0.45 V. Also, the AC exhibits an excellent energy density of ~ 19.3 Wh kg^−1^ and power density of ~ 277.92 W kg^−1^, respectively, in the same operating potential window. It has also shown very good capacitance retention capability even after 5000th cycles. The fabricated supercapacitor shows a good energy density and power density, respectively, and good retention in capacitance at remarkably higher charging/discharging rates with excellent cycling stability. Henceforth, bio-waste Kusha grass-derived activated carbon (DP-AC) shows good promise and can be applied in supercapacitor applications due to its outstanding electrochemical properties. Herein, we envision that our results illustrate a simple and innovative approach to synthesize a bio-waste Kusha grass-derived activated carbon (DP-AC) as an emerging supercapacitor electrode material and widen its practical application in electrochemical energy storage fields.

**Supplementary Information:**

The online version contains supplementary material available at 10.1186/s11671-021-03545-8.

## Introduction

In recent years, much attention has been paid toward the development of the promising sustainable energy storage models which includes conversion and storage devices in pursuit of the global energy exigencies [1–3]. Energy storage devices such as batteries and supercapacitors play very significant, efficient, and affordable roles in the generation of renewable and sustainable energy sources and are viable alternatives to traditional non-renewable options. Supercapacitors have emerged as most promising sustainable energy storage devices due to long cycle life, high power density, and ultra-fast charging/discharging time [4–8]. Moreover, due to the burgeoning research area of carbon-based nanomaterials such as graphene, nanotubes, nanodots, and quantum dots, the intensive development of supercapacitor energy storage devices has also been increased [9–11]. Studies reveal many research works have been focused on the synthesis of materials and their composites with other hybrids demonstrating high capacitance, wide potential window, lesser impedance, and good capacitive retention [12]. Also, the fabrication of electrodes has pulled wide attention with high mass loading of activated material and mass-to-the current collector ratio [13, 14].

Supercapacitors based on carbon materials have been widely studied and offer wide potential windows leading to the high energy density in the presence of organic electrolytes [15, 16]. Therefore, carbon-based supercapacitors show high resistance and low capacitance. But, organic electrolytes are toxic, flammable, and comparatively expensive [15]. Carbon nanotubes, graphene, etc., are exorbitant to some extent on the methods of preparation and availability of primal materials and restrain their large-scale applications. Therefore, research dealing with several changes for different carbon materials has been performed to increase the potential window, supercapacitance performance, and lesser impedance with eco-friendly, cost-effective, and easy-to-employ method [17, 18].

Activated carbon having high surface area, ample functional associates, and sufficient porosity has been extensively used for adsorption, gas storage, gas separation, catalyst support, solvent decoloring, solvent recovery, electrodes, and supercapacitors over the past few decades. Its porous structure and other properties such as high surface area, pore volume, presence of different types of functional groups, and distribution of pore sizes play a crucial role in the absorption-related applications of the activated carbon [18]. Depending upon the pore size, activated carbon can be used in different application fields such as micropores are used in adsorption of the smaller molecule, while mesopores are extensively used in the adsorption of the larger molecules [19, 20].

Many factors affect the properties of the activated carbon such as raw materials, synthesis route, activating reagent, and environmental conditions during the activation process. AC is synthesized by adopting different synthesis routes and precursors which are bio-waste/naturally available such as coconut shells [21], neem [22], corn starch [23], recycled waste paper [24], scrap tires [25], and banana fiber [26]. AC is mainly synthesized through physical and chemical activation processes [19]. Usually, the first one primarily involves carbonization and further activation in an inert atmosphere or the presence of gas such as CO_2_ or oxidizing agents [27], whereas the chemical activation process first includes the development of the porous structures by adding activating agents such as ZnCl_2_, NaOH, H_3_PO_4_, and KOH [12, 28–30]. According to studies, ZnCl_2_ is not a much preferable active agent due to environmental concerns and incompetent recuperation. Therefore, the AC activated through ZnCl_2_ has not been suitable for pharmaceutical and agro-food industrial purposes as they have a probability to contaminate the results [20]. Among other chemical reagents, KOH has been widely used as it results in ACs with high surface area and well-defined pores. Gonzalez et al. reported the KOH activation of cherry stones resulting in microporous ACs with large capacitances [31]. Yushin et al. synthesized the wood sawdust-based ACs through hydrothermal carbonization, followed by activation from KOH, and studied its supercapacitor performance [32]. Ranganathan et al. illustrated the synthesis of ACs from the waste paper using KOH as an activating agent. It exhibits a specific capacitance of 180 F g^−1^ in the KOH electrolyte [24]. He et al. used a rapid microwave heating technique to synthesize ACs from cokes and studied the KOH–coke mass ratio and activation time [33]. The activating agents play a vital role during the process such as dehydrating agents prevent the progression of several intermediate products. It also increases the density of porous sized structures and reduces the activation time as well as temperature [24, 34, 35].

In the present work, Kusha grass (*Desmostachya bipinnata*) has been used as an eco-friendly, cost-effective, and plenty of carbonaceous precursors for the synthesis of activated carbon. To synthesize AC, a chemical process involving KOH as an activating agent has been adopted due to its better reliability. The as-synthesized AC material has been characterized through UV–visible, Fourier transform infrared, and Raman spectroscopy. Further, to access the validation of the structural features, the as-synthesized material has been characterized by scanning electron microscopy (SEM), energy-dispersive spectroscopy (EDAX), TEM, and XRD techniques. For the application purpose, electrochemical and galvanometric charge techniques have been adopted by following a bit modification in electrode with a three-electrode system. Due to the reliability of the GC technique, it has been used for other calculations such as supercapacitance, energy density, and current density. It reveals that prepared AC exhibits excellent supercapacitance properties due to well-defined porous features. Henceforth, this study endows the first of its kind dealing with the fabrication of highly capacitive activated carbon (AC) using a bio-waste Kusha grass (*Desmostachya bipinnata*).

## Methods

### Materials

Kusha grass (*Desmostachya bipinnata*; DP) was collected from the botanical garden of the BHU campus, Varanasi, India, while potassium hydroxide (KOH), glassy carbon electrode (GCE), and alumina powder were procured from Sigma-Aldrich. Aqueous solutions used throughout all the experiments were prepared by using deionized water (DI > 18 MΩ cm^−1^, Millipore Q system).

Different analytical techniques have been employed to characterize the as-synthesized samples. To access the structural features and crystalline properties of the as-synthesized activated carbon, powder X-ray diffraction was performed on a PANalytical X-ray diffractometer using CuK_α_ radiation (*λ* = 1.540 Å) at 2*θ* ~ 10°–80°. The microstructures and surface morphologies of the as-synthesized material were studied by a transmission electron microscope (TEM, TECHNAI G^2^ operated at 200 kV) and a scanning electron microscope (Dual FIB: FEI Nanolab operated at 200 kV). TEM sample was prepared by drop-casting of suspension of DP-AC powder over a carbon-coated grid followed by ultrasonic suspension in DI water. Further, some additional structural features of the as-synthesized activated carbon were confirmed by Raman spectroscopy. Raman scattering measurement was carried out with a 532-nm He–Ne laser excitation using a Raman spectrometer (Renishaw inVia, UK). Moreover, the Fourier transform infrared (FTIR) spectrometer (Bruker ALPHA II) was employed to investigate the presence of the functional groups attached to the as-synthesized sample. The optical properties were evaluated by UV–visible light absorption spectroscopy recorded through fluorescent lamps (*λ* = 365 nm) (PerkinElmer, Lambda 25). The surface area and pore size distribution of the as-prepared activated carbon sample were measured by employing liquid nitrogen adsorption/desorption analysis adopting an automatic Brunauer–Emmett–Teller (BET) method (micromeritics FlowPrer 060, Gemini VII, USA).

### Synthesis of Activated Carbon

**Fig. 1:**
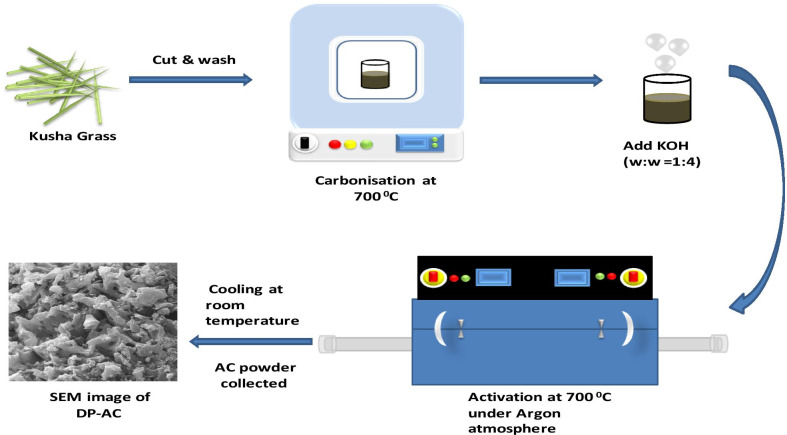
Illustration of the strategic pathway for the synthesis of activated carbon from Kusha grass (*Desmostachya bipinnata*)

Briefly, Kusha grass (DP) was cut and washed gently several times with DI water until the supernatant turned out to be colorless. DP was kept in an oven at 100 °C for 5 h and further carbonized for 2 h in a muffle furnace uphold at 700 °C. For activation, it was mixed in proven KOH (w/w 1:4) with the help of mortar–pestle, and subsequently, the homogeneous mixture was collected. Further, it was held in a tubular furnace at 700 °C for 2 h in argon ambiance. The mixture was further cooled to room temperature, and the as-received mixture was washed several times with DI water till pH attained a value of ~ 7. Finally, we got the product as activated carbon and kept it safe in a vessel for further experiments and measurements. The overall process is illustrated in Fig. 1.

### Electrode Preparation

A glassy carbon electrode (GCE) of diameter ~ 0.3 cm was polished with alumina slurry (0.05 μm). In the next step, DI water was used for cleaning the surface of the GCE. It was washed 3–4 times with DI water and further sonicated for 15–20 min in DI water and ethanol. For the deposition of AC, 1 mg of active material (AC) was dissolved in DI water (1 mL) and sonicated for 15 min. Further, 10 μL of the prepared solution was dropped cast over GCE, with the help of micropipette, and dried in lamp light without any nearby contact for the prevention of contamination.

### Electrochemical Testing

Electrochemical experiments have been performed on the CHI-660C multichannel workstation with a three-electrode system using pt wire, Ag/AgCl, and glassy carbon electrode as a counter, reference, and working electrode, respectively. An alkaline 6 M KOH aqueous electrolyte was applied to carry out measurements. Cyclic voltammetry at different scan rates (10–200 mV s^−1^) was performed with the sweeping potential window − 0.35 V to + 0.45 V. The different electrochemical parameters have been accessed by using the following equations [35–37].

The specific capacitance has been evaluated as

1 $$C_{{\text{s}}} = \frac{{I_{{{\text{Avg}}}} }}{\nu \times m}$$

where $I_{{{\text{Avg}}}} = I_{\max } - I_{\min }$ and also *m* and *ν* represent the mass of the loading material (g) and scan rate (v/s), respectively. Moreover, *C*_s_ has a unit of F g^−1^.

Since the galvanometric charge–discharge (GCD) technique is more reliable and gives more accurate results, we have adopted the GCD technique for further calculations. We calculated the specific capacitance by using the equation

2 $$C_{{\text{s}}} = \frac{{I \times\Delta t}}{{\Delta V \times m}}$$

where *I*, ∆*t*, ∆*V*, and *m* represent the current (*A*), discharging period (*s*), voltage windows (*V*), and mass of the loading material (*g*), respectively.

Further, energy density (*E*) and power density (*P*) have been deduced through the equations

3 $$E = \frac{{c_{{\text{s}}} { } \times\Delta V^{{2{ }}} }}{7.2}\quad { }\left( {\text{Wh/kg}} \right)$$

4 $$P = \frac{E \times 3600 }{{\Delta t}} \quad \left( {{\text{W}}/{\text{kg}}} \right).$$

## Results and Discussion

### X-ray Diffraction

**Fig. 2:**
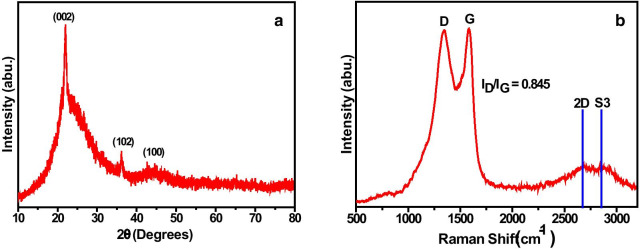
**a** XRD pattern and **b** Raman spectrum of as-synthesized activated carbon

To access information regarding the graphitization of as-synthesized activated carbon material, the XRD technique has been adopted. The XRD profile (Fig. 2a) clearly shows the characteristic peaks of activated carbon material at 22° and 43° [38, 39]. The intensity and position of peaks unveil the low degree of graphitization, regularities of the crystalline structure, and formation of K_2_CO_3_ (2*θ* = 36.52°) as an intermediate product [40–42]. Further, the obtained characteristic peaks can be assigned to the reflection planes (002) and (100) for the DP-AC. The broad peak in the spectrum stipulates the amorphous carbon, while the sharpness of the peak assigned at 22° shows the increased translational order in the carbon sample at high temperature. These results confirm the successful formation of as-synthesized AC material.

### Raman Analysis

Further, the material has been characterized by Raman spectroscopy, a most pronounced technique to characterize various carbonaceous materials. There are two most intense peaks observed in the Raman spectrum of activated carbon material positioned at 1346 cm^−1^ (D peak) and 1587 cm^−1^ (G peak) as shown in Fig. 2b. The D peak is the characteristic of lattice defects, edge imperfections, unkempt alignment, and low-symmetry graphitic structure in activated carbon material [43], and the second peak, i.e., G peak, demonstrates the occurrence of C=C stretching vibrations [10]. Besides, it exhibits two more bands at higher wavenumbers 2678 cm^−1^ (2D) and 2840 cm^−1^ (S3) due to the overtone of carbon and reveals the presence of few-layered carbon material and the graphitic nature of activated carbon material [44].

Further, the degree of graphitization has been calculated through the equation

6 $$R = \frac{{I_{{\text{D}}} }}{{I_{{\text{G}}} }}$$

where *R*, *I*_D,_ and *I*_G_ represent the degree of graphitization, the intensity of the D-peak positioned at 1346 cm^−1^, and the intensity of the G-peak positioned at 1587 cm^−1^, respectively. After the calculation, the value of *R* has found to be ~ 0.84, which refers to a higher index of graphitization to some extent [10].

### Morphological Characterization and Energy-Dispersive X-Ray (EDX) Analysis

**Fig. 3:**
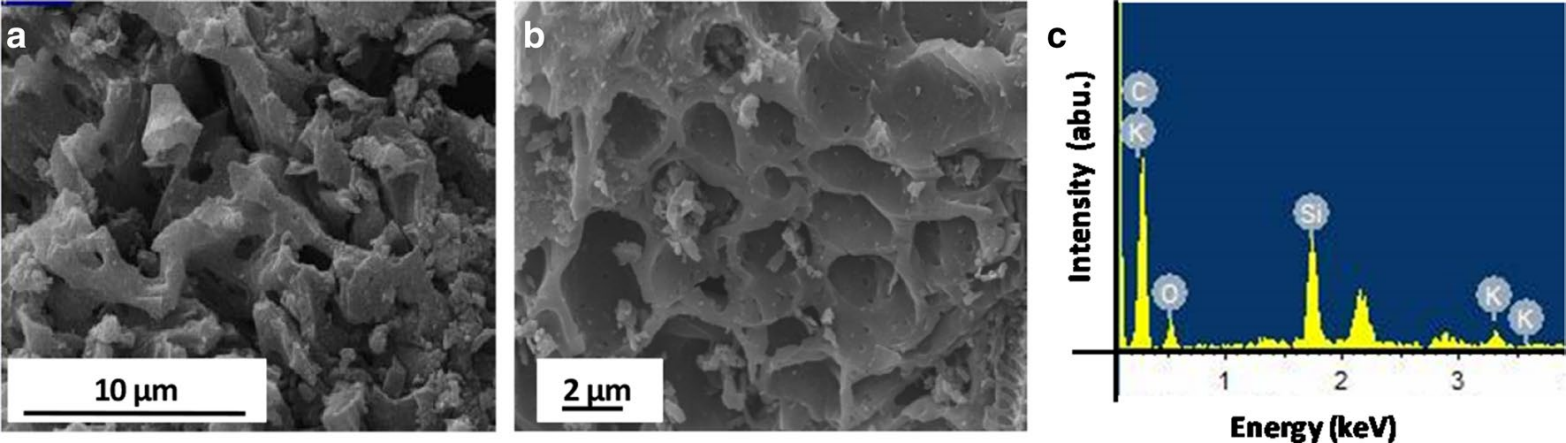
**a** SEM image (bar scale 10 μm), **b** SEM image (bar scale 2 μm), and **c** EDAX profile of as-prepared sample

To explore the microstructural features and surface morphology of the as-synthesized material, scanning electron microscopy (SEM) images as shown in Fig. 3a, b have been analyzed. The morphology suggests the presence of large irregular pores in the as-synthesized DP-AC. The occurrence of irregular and disordered pore structures on the surface accounts for the violent attack of reagent KOH. DP-AC pores developed during pyrolysis are crucial to enhance the surface area and pore volume of the activated carbon by promoting the diffusion of KOH molecules into the pores and thereby increase the carbon reaction, which is here assumed to generate additional pores in the AC. The large pore size structure on the surface of the activated carbon material has been advantageous for the charge storage applications like supercapacitors. Moreover, the elemental analysis of the as-synthesized activated carbon material (Fig. 3c) has been done via energy-dispersive X-ray spectroscopic technique and divulges the existence of carbon, oxygen, and potassium elements in it.

### Transmission Electron Microscopy (TEM) and Particle Size Distribution

**Fig. 4:**
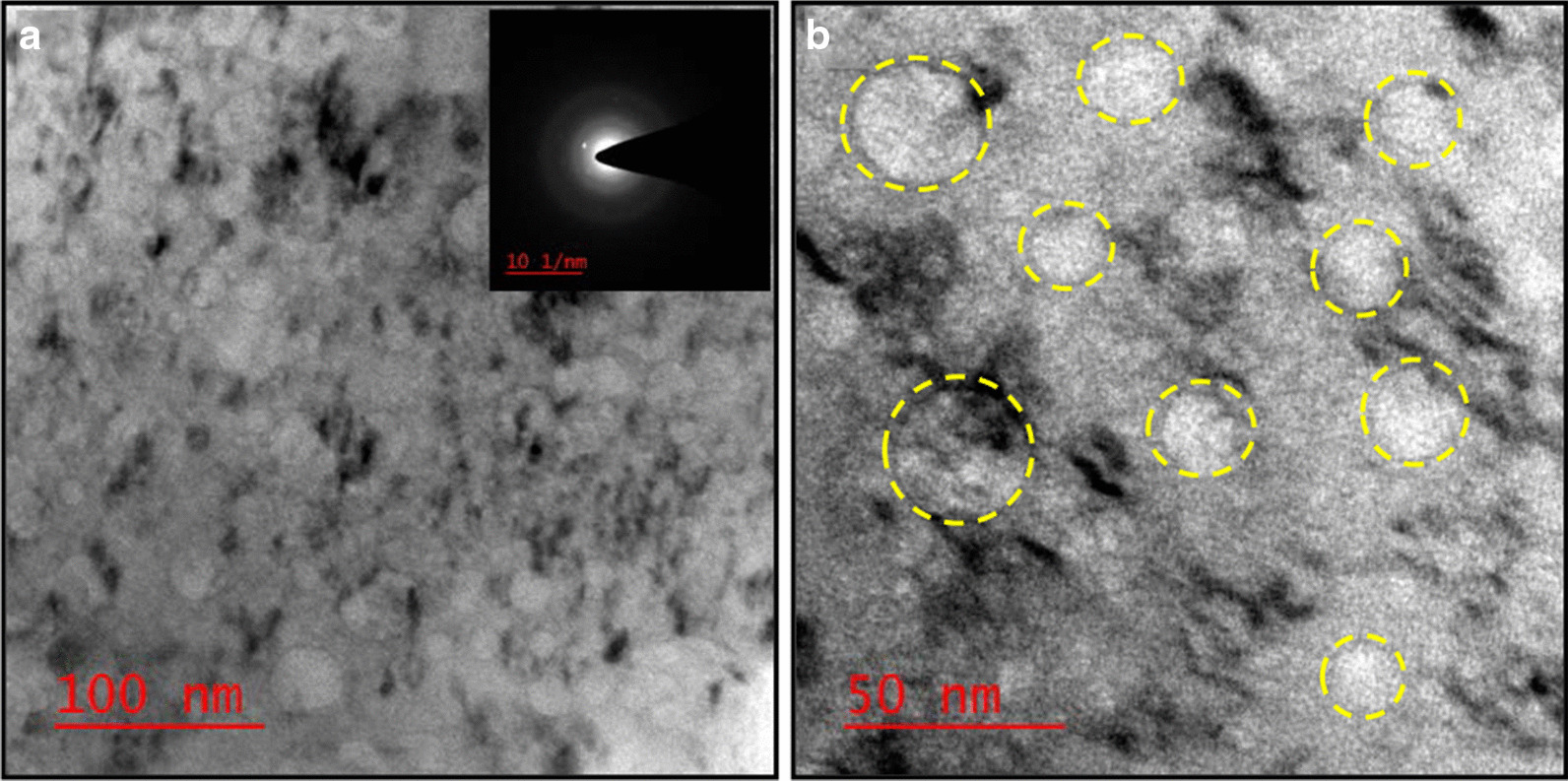
**a** TEM image (bar scale 100 nm) (inset: SAED pattern), **b** TEM image showing various sizes of porous structures (bar scale 50 nm) of the as-synthesized activated carbon material

Further, to authenticate more structural information, crystal quality dimensions of the prepared sample, transmission electron microscopy (TEM) has been performed. TEM images infer the presence of several pore size structures that can be seen as transparent sites (circled with yellow color) in Fig. 4a, b. Moreover, the SAED pattern reveals the amorphous nature of the activated carbon material as shown in the inset of Fig. 4a.

### UV–Visible Light Absorption and FTIR Analysis

**Fig. 5:**
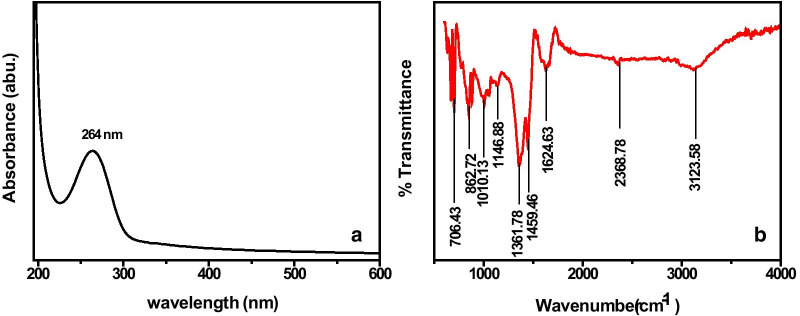
**a** UV–visible spectrum and **b** FTIR spectrum, of the Kusha grass-derived as-synthesized activated carbon sample

The UV–visible absorption spectrum of the as-synthesized activated carbon material has been recorded and is represented in Fig. 5a. It possesses a characteristic absorption peak at 264 nm due to the electronic transitions between the bonding and antibonding *π*-orbitals.

The surface chemical properties of as-synthesized activated carbon material have been analyzed by FTIR spectroscopy and are shown in Fig. 5b. It gives details of the associated functional groups in the activated carbon material. The appearance of an absorption band at 3115 cm^−1^ and a small peak at 2368.78 cm^−1^ owes to −OH stretching vibration of hydroxyl functional groups [10, 45, 46]_._ The peak at 1624.63 cm^−1^ is associated −C=C stretching of the aromatic rings_,_ which may be formed because of the decomposition of C–H bonds to form a more stable –C=C group at higher activation temperature [47]. The strong absorption bands at 1459.46, 1361.78, 1146.88, 1010.13, and 862.72 cm^−1^ confirm the presence of –C–C (conjugated with –C=C),  –CH_3_ vibration, C–N stretching, C–O stretching modes of ester, and  – C–O symmetric stretching [39, 43, 47–49] in the as-synthesized activated carbon material, respectively. Moreover, the strong absorption peak at 706.43 cm^−1^ attributes to –C=C bending in the as-synthesized DP-AC.

### BET Analysis

#### N_2_ Adsorption and Desorption Isotherms

**Fig. 6:**
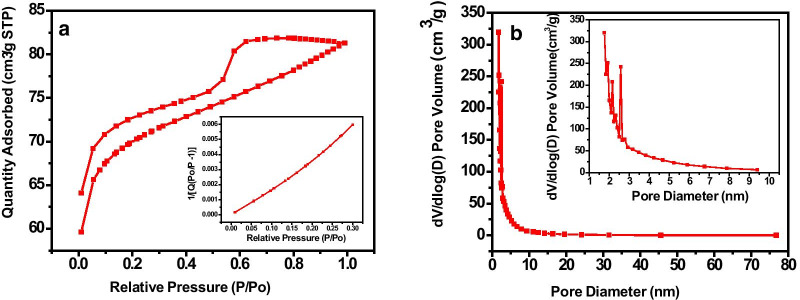
**a** N_2_ adsorption–desorption analysis isotherm (inset showing a relation between relative pressure (*P*/*P*_0_) versus 1/[*Q*(*P*_0_/*P − *1)]), **b** BJH plot; pore size distribution plot of activated carbon materials (inset shows magnified view demonstrating the existence of mesopores for DP-AC)

The porosity in the carbon material has been generated with activation through a reagent KOH during the synthesis. The pore structure and surface area are regarded as significant factors for the supercapacitor or ultracapacitor ability of the materials [11]. The as-synthesized DP-AC has been analyzed by N_2_ adsorption–desorption test based on the BET principle for pore structure characteristics and surface area. Figure 6a depicts the nitrogen adsorption–desorption isotherm of DP-AC activated through K_2_CO_3_. The shape of N_2_ adsorption–desorption isotherm is assumed as a mixed-type isotherm, i.e., it includes isotherms II and IV. Type II isotherm assumed as the combination of type I and II isotherms is indicative of the existence of microporous nature. The initial part (concave shape) demonstrates the complete coverage of monolayer and further absorption of multilayer [11]. Hence, type II isotherm reveals good agreement in microporous as well as microporous structures. At the relatively higher pressure, the graph uptake remarkably signifies type IV isotherms having a hysteresis loop. Moreover, the type IV isotherm ascribes to monolayer and multilayer adsorption accompanying capillary condensation that takes place in tapered slit-like pores. Further, the surface area and pore size diameter have been evaluated using the BET equation (Eq. 7).

7 $$\frac{1}{{Q\left( {\left( {\frac{{P_{0} }}{P}} \right) - 1)} \right)}} = \frac{1}{{Q_{m} C}} + \frac{C - 1}{{Q_{m} C}}\left( {\frac{P}{{P_{0} }}} \right)$$

where (*P*/*P*_*0*_) represents the relative pressure and *Q*, *Q*_*m*_, and *C* represent the weight of gas adsorbed, adsorbate as a monolayer, and BET constant, respectively. The surface area parameters such as BET surface area, micro- and mesopore surface area, total pore volume, micro- and mesopore volume, and average pore diameter of DP-AC have been deduced and are summarized in Additional file 1: Table S2 of Supplementary Information section.

### The Pore Size Distribution of the DP-AC

Figure 6a (inset) shows a straight line for the quantity absorbed vs relative pressure (*P*/*P*_0_), which is a good agreement for the calculation of total surface area. Figure 6b shows the pore size distribution against the differential volume. To investigate the pore size distribution, BJH analysis has been performed. The average pore size diameter and width of the prepared activated carbon sample have been found as ~ 3.3 nm and ~ 2.3 nm, respectively. The corresponding average pore volume has been 0.126 cm^3^ g^−1^. Figure 6b (inset) depicts the magnified view of the BJH differential volume and distribution of pore size diameter for DP-AC. The BET isotherm curve reveals the surface area to possess a value of ~ 738.56 m^2^ g^−1^, which infers the existence of meso- and macropores in the as-synthesized sample. Materials having a high surface-to-volume ratio and abundance of mesopores stimulate sufficient charge storage (energy density) and fast charge transfer kinetics (power density), respectively, and are crucially prominent in advanced energy storage.

### Electrochemical Analysis

Cyclic voltammetry (CV), galvanostatic charge–discharge (GCD), and electrochemical impedance spectroscopy (EIS) analyses have been used to observe the electrochemical performance of DP-AC for supercapacitor. All of the investigations have been performed with a three-electrode system in 6 M KOH as an electrolyte solution.

**Fig. 7:**
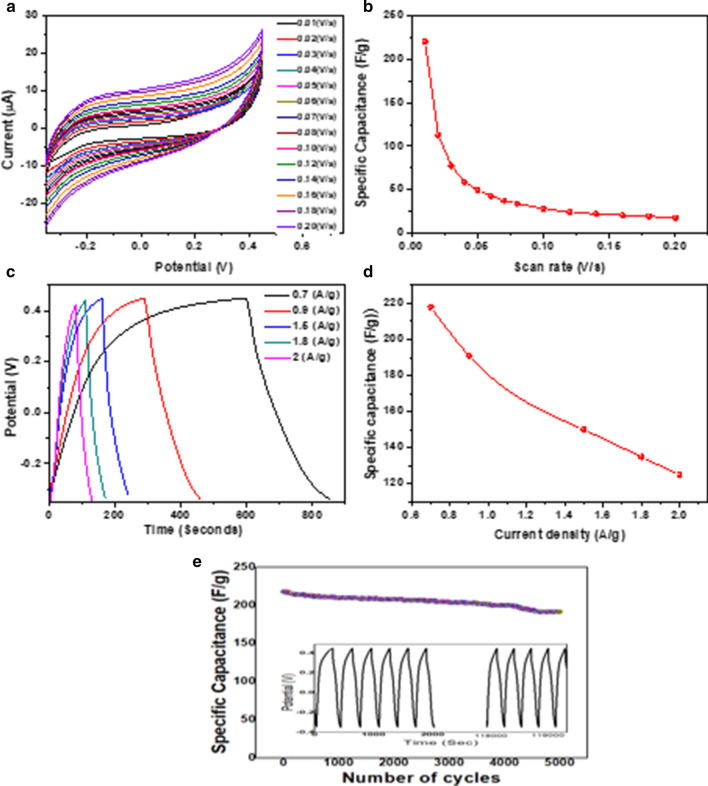
**a** Cyclic voltammogram (CV) at different scan rates, **b** specific capacitance through CV, **c** galvanostatic charge–discharge curves at different current densities, **d** specific capacitance through GCD, **e** cyclic stability of the as-synthesized DP-AC electrode material up to 5000th cycle with initial and final cycles at a current density of 0.7 A g^−1^

The electrochemical performance evaluation using CV is summarized in Fig. 7a. The figure depicts CV curves at varied scan rates in the range of 10–200 mV s^−1^ within the potential window of 0.35 V to + 0.45 V and shows the rectangular shape of the cyclic volumetric curve of AC. Figure 7b displays a common characteristic of an electrochemical energy storage device. A low scan rate exhibits a higher value of specific capacitance than at a higher rate as at a low scan rate, the ions in the electrolyte can diffuse into the accessible pores of the electrode, allowing good interaction between the ions and pores of the electrode to occur. At higher rates, poor accessibility or lesser availability of time for hydroxyl ions to get transferred from the electrolyte to the electrode surface, and thus ions cause the specific capacitance to decrease [50, 51]. We have observed maximum specific capacitance at 10 V s^−1^ scan rate, while the scan rate is decreased from 160 to 10 V s^−1^. High surface area and high porosity play a crucial role to possess high capacitance. Moreover, the closed rectangular shape of the CV attributes to the optimization of combined micropore and mesopore volume as well as good electrical conductivity. Henceforth, the highest specific capacitance, *C*_SP_ as ~ 220.70 F g^−1^ has been found at 10 mV s^−1^ for DP-AC calculated using Eq. 1.

These excellent performances of DP-AC attribute to its porous sheet-like structure which plays a vital role for electrolyte ions enabling rapid charge transport and storage.

Furthermore, electrochemical capacitance and columbic efficiency have been deduced from the electrochemical performance of the electrode material by employing the galvanostatic charge–discharge technique in a fixed potential window at controlled current situations at different current densities 0.7, 0.9, 1.5, 1.8, and 2 A g^−1^. It displays a nearly triangular-shaped galvanometric profile (Fig. 7c), signifying the EDLC characteristic in the DP-AC electrode. The maximum *C*_SP_ has been deduced as 218 F g^−1^ at a current density of 0.7 A g^−1^ for DP-AC from Eq. 2. Figure 7d depicts the variation of specific capacitance with current density [52–54]. As the current density increases gradually, the specific capacitance decreases slowly. It is known that when the charging current becomes faster, it is difficult for the electrolyte ions to rapidly diffuse into the corresponding pores of the electrode material. Moreover, since the cyclic stability of the material is a crucial parameter for practical uses of the supercapacitor, the cyclability of the DP-AC electrode material has been carried out. Figure 7e shows that ~ 88% of the initial specific capacitance is retained and suggests its ability for fast charging and discharging without hardly any degradation even after the 5000th cycle [50, 53, 54] and in turn confirms the durability of the as-prepared material.

**Fig. 8:**
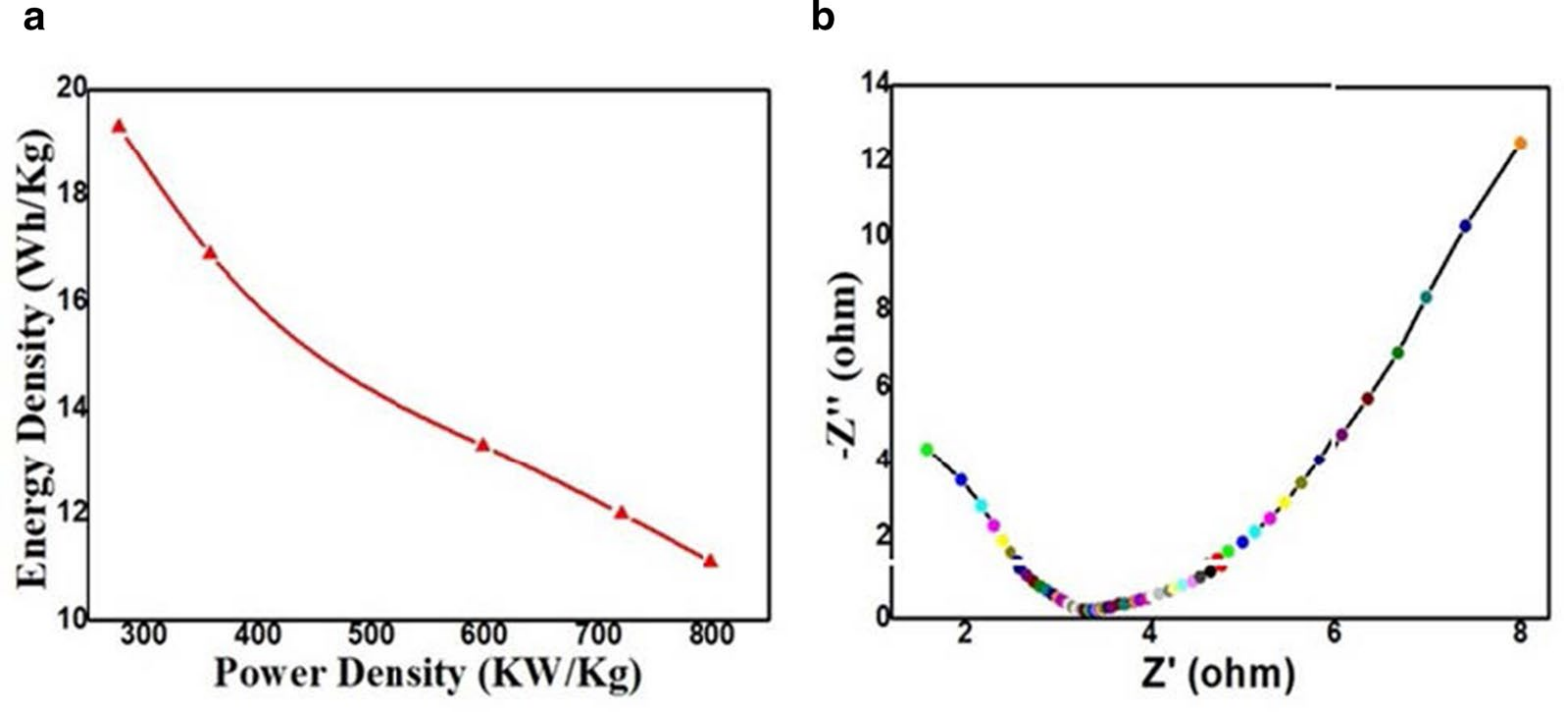
**a** Ragone plot for the GCD capacitor and **b** Nyquist plot of impedance for as-synthesized DP-AC

To further validate the performance of the as-synthesized DP-AC material for energy storage applications in practical life, energy and power densities are regarded as two vital parameters and have been deduced from the charge/discharge profile using Eqs. 3 and 4. It exhibits a maximum energy density of 19.3 Wh kg^−1^ with a reasonably good power density of 277 W kg^−1^ as evaluated in − 0.35 V to + 0.45 V range and is shown in Fig. 8a. Thus, in accord with the Ragone plot, we have developed a supercapacitor with enhanced energy density and without loss in power density which can be used practically. Also, remarkable *C*_SP_ in a wide potential window demonstrates a sufficient increase in the energy density of as-synthesized DP-AC. Some extended calculations related to supercapacitor performance have been deduced and thus displayed in Additional file 1: Table.S1.

Electrochemical impedance spectroscopy (EIS) technique has been adopted to investigate the interfacial properties such as capacitive and resistive characters of the as-synthesized material at the electrode–electrolyte interface [52] through Nyquist plot (Fig. 8b) and Bode plot (Additional file 1: Fig.S1). Fig. 8b illustrates the Nyquist plot between – Z” (imaginary part) and Z’ (real part) measured in the frequency range of 0.01 Hz to 0.1 GHz at an AC amplitude of 5 mV in the open circuit potential. It shows electric resistance of 1.58 Ω along a small diameter of semicircle confirming high conductivity and low internal resistance. The intersection between the curve and horizontal axis represents the total electric resistance of the device. The diameter of the semicircle at high frequency owes to the charge transfer resistance between electrode material and electrolyte, and tail slope at low frequency attributes to the ionic diffusion rate in the electrolyte [55–57]. Therefore, an electrode with as-synthesized DP-AC suits well for supercapacitor applications.

## Conclusion

In summary, a very new facile and low-cost synthesis strategy has been illustrated in the present study for the development of activated carbon material with well-developed pores and high surface area from a natural precursor Kusha grass. It demonstrates a sustainable, eco-friendly, easy-to-employ, without any complex post-synthesis procedure for the energy storage application like a supercapacitor. The fabricated DP-AC with excellent properties has been used as an electrode material for electrochemical supercapacitors. The route enables a bit of modification of the electrode system with a loading of 1 × 10^−5^ g DP-AC sample and exhibits a significantly high collector current–mass ratio. The highest specific capacitance has been observed with the CV technique as 220.70 F g^−1^ and with GCD as 218 F g^−1^ in a wide operating potential window, which is comparably higher than reported works on the ground of green synthesis (Table 1). The fabricated supercapacitor shows a good energy density and power density as 19.3 Wh kg^−1^ and 277.92 W kg^−1^, respectively, and good retention in capacitance at remarkably higher charging/discharging rates with excellent cycling stability. Henceforth, bio-waste Kusha grass-derived activated carbon (DP-AC) with optimal electrochemical performance can be explored successfully at a real scale, and electrochemical electrical energy store devices with Kusha grass-based AC material may be realized in a short period.

**Table 1:** Comparison of the present work with other related works based on different natural bio-waste precursors

S. nos.	Bio-waste carbon source	Activation method	BET surface area (m^2^ g^−1^)	Specific capacitance (F g^−1^)	Electrolyte	References
1.	Oil palm kernel shell	Steam	727	210	1 M KOH	[58]
2.	Corn cob	KOH	42	99	6 M KOH	[36]
3.	Rice straw	H_3_PO_4_	396	112	1 M H_2_SO_4_	[59]
4.	Rice husk	H_3_PO_4_	1490	112	1 M Na_2_SO_4_	[60]
5.	Scrap waste tire	H_3_PO_4_	510	93	6 M KOH	[25]
6.	Recycled waste paper	KOH	416	180	6 M KOH	[24]
7.	Banana fiber	ZnCl_2_	1097	74	1 M Na_2_SO_4_	[26]
8.	Kusha grass (*Desmostachya bipinnata*)	KOH	738.56	218	6 M KOH	Present work

## Supplementary Information

**Additional file 1.** Supporting file comprises different significant suparcapacitor parameters (Table S1), Nitrogen adsorption-desorption data (Table S2) and Bode plot (Fig. S1) of the Kusha grass derived as-synthesized activated carbon material. **Table S1.** Different significant supercapacitor parameters of the supercapacitor based on Kusha grass-derived as-synthesized activated carbon material. **Table S2.** Nitrogen adsorption-desorption data of the as-synthesized DP-AC. **Figure S1.** Bode plot of as-synthesized DP-AC electrode materials.

## Data Availability

The used datasheets and materials are available from the corresponding authors on reasonable request.

## Abbreviations

DP: *Desmostachya bipinnata*
AC: Activated carbon
KOH: Potassium hydroxide
GCE: Glassy carbon electrode
XRD: X-ray powder diffraction
TEM: Transmission electron microscopy
SEM: Scanning electron microscopy
EDAX: Energy-dispersive X-ray spectroscopy
FTIR: Fourier transform infrared spectroscopy
BET: Brunauer–Emmett–Teller
CV: Cyclic voltammetry
GCD: Galvanostatic charge–discharge
EIS: Electrochemical impedance spectroscopy
