# Supplementary material for: In Situ Fabrication of Activated Carbon from a Bio-Waste Desmostachya bipinnata for the Improved Supercapacitor Performance
Source: Nanoscale Res Lett. 2021 May 13;16:85. doi: 10.1186/s11671-021-03545-8 (PMC8119520; doi:10.1186/s11671-021-03545-8)
Supplement: Supplementary file 1 — Additional file 1. Supporting file comprises different significant suparcapacitor parameters (Table S1), Nitrogen adsorption-desorption data (Table S2) and Bode plot (Fig. S1) of the Kusha grass derived as-synthesized activated carbon material. Table S1. Different significant supercapacitor parameters of the supercapacitor based on Kusha grass-derived as-synthesized activated carbon material. Table S2. Nitrogen adsorption-desorption data of the as-synthesized DP-AC. Figure S1. Bode plot of as-synthesized DP-AC electrode materials. [file 11671_2021_3545_MOESM1_ESM.doc]

**Supporting Information**

**In-Situ fabrication of activated carbon from a bio-waste Demostachya Bipinnata for the Improved Supercapacitor performance**

Gopal Krishna Gupta^1^, Pinky Sagar^2^, Sumit Kumar Pandey^2^, Monika Srivastava^3^, A K Singh^4^, Jai Singh^5^, Anchal Srivastava^2^, S. K. Srivastava^2^, *, Amit Srivastava^1^, *

^1^Department of Physics, TDPG College, VBS Purvanchal University, Jaunpur - 222001, India

^2^Department of Physics, Institute of Science, Banaras Hindu University, Varanasi - 221005, India

^3^School of Materials Science & Technology, Indian Institute of Technology (BHU), Varanasi - 221005, India

^4^School of Physical Sciences, Jawaharlal Nehru University, New Delhi- 110067, India

^5^Department of Pure & Applied Physics, Guru Ghasidas Vishwavidyalaya , Bilaspur- 495009, India

* Corresponding Authors

Emails: [amitrac@gmail.com](mailto:amitrac@gmail.com), sanjay_itbhu@yahoo.com

**Table.S1. Different significant supercapacitor parameters of the supercapacitor based on Kusha grass-derived as-synthesized activated carbon material.**

| **S.No.** | **Current Density (A/g)** | **Specific Capacitance (F/g)** | **Discharge Time (Seconds)** | **Energy Density (Wh/kg)** | **Power density (W/kg)** |
| --- | --- | --- | --- | --- | --- |
| i | 0.7 | 218 | 250 | 19.3 | 277.92 |
| ii | 0.9 | 191 | 170 | 16.9 | 357.8 |
| iii | 1.5 | 150 | 80 | 13.3 | 598.5 |
| iv | 1.8 | 135 | 60 | 12.0 | 720 |
| v | 2 | 125 | 50 | 11.1 | 799 |

**Table.S2. Nitrogen adsorption-desorption data of the as-synthesized DP-AC.**

| i | BET surface area | ~ 738.56 m²/g |
| --- | --- | --- |
| ii | Micropore surface area | ~ 691.462 m²/g |
| iii | Mesopore and external surface area | ~ 47.098 m²/g |
| iv | Total pore volume | ~ 0.125769 cm³/g |
| v | Micropore volume | ~ 0.086751 cm³/g |
| vi | Mesopore volume | ~ 0.039018 cm³/g |
| vii | Average pore diameter | ~3.3 nm |
| viii | Average pore size width | ~2.3 nm |


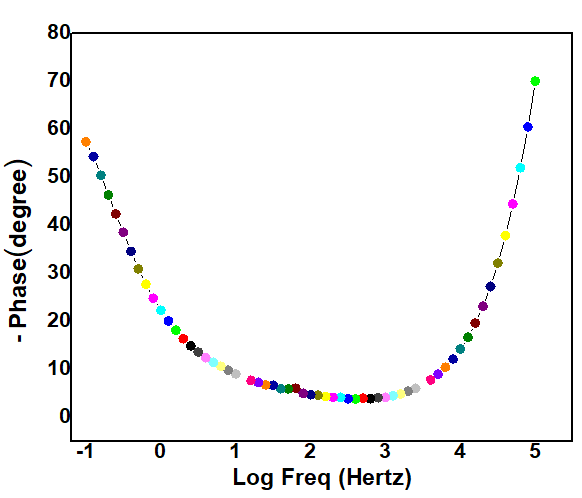


**FigureS1: Bode plot of as-synthesized DP-AC electrode materials.**
